# Platelet Releasate Reprograms Synovial Macrophages In Vitro: A New Approach in the Treatment of Hemophilic Synovitis

**DOI:** 10.3390/ijms262110616

**Published:** 2025-10-31

**Authors:** Paula Oneto, María Eulalia Landro, Martin Manuel Ledesma, Julia Etulain, Carla Daffunchio, Guillermo Cambiaggi, Mirta Schattner, Andrea Emilse Errasti, Horacio Caviglia, Eugenio Antonio Carrera Silva

**Affiliations:** 1 Institute of Experimental Medicine (IMEX), CONICET, National Academy of Medicine, Buenos Aires C1425AUM, Argentina; onetopaula7@gmail.com (P.O.); mmledesma88@gmail.com (M.M.L.); juliaetulain@hotmail.com (J.E.); mschattner@hotmail.com (M.S.); 2 Department of Traumatology, Juan A. Fernández Hospital, Buenos Aires C1425AGP, Argentina; mlandro@gmail.com (M.E.L.); carladaffunchio@gmail.com (C.D.); gcambiaggi@gmail.com (G.C.); 3 Haemophilia Foundation, Buenos Aires C1425BWE, Argentina; hacbagenova@gmail.com; 4 School of Medicine, Argentine Catholic University, Buenos Aires C1107AAZ, Argentina; 5 Institute of Pharmacology, School of Medicine, University of Buenos Aires, Buenos Aires C1053ABH, Argentina; andreaerrasti@gmail.com

**Keywords:** chronic hemophilic synovitis, platelet-rich plasma, macrophages, NETs

## Abstract

Chronic hemophilic synovitis (CHS), driven by hemosiderin-laden macrophages from recurrent hemarthrosis, is a major cause of joint damage in hemophilia. Platelet-rich plasma (PRP) is a promising regenerative therapy for joint diseases. This study investigated PRP’s ability to modulate macrophage polarization from a pro-inflammatory (M1) to a pro-resolving, tissue-repairing (M2) phenotype in CHS. We analyzed synovial fluid (SF) from CHS patients (N = 22), both pre- and post-PRP treatment. Ex vivo analysis revealed a predominant M1 profile with an increased proportion of CD11^+^CD14^+^CD64^hi^ compared with CD206^+^ or CD163^+^ M2 macrophages in CHS SF. In vitro experiments showed that CHS SF skewed monocyte-derived macrophages toward an M1 inflammatory program, evaluated by flow cytometry, qPCR, and ELISA. However, adding PRP significantly modulated the pro-inflammatory macrophage program, promoting an M2 tissue repair profile. Furthermore, a random forest machine learning algorithm, applied to public scRNAseq data, confirmed PRP’s macrophage reprogramming effect. Functional assays also showed increased TGF-β secretion and macrophage fusion when challenged with neutrophil extracellular traps (NETs). A small patient follow-up cohort treated with intra-articular PRP showed similar results, including normalization of cellular content and reduced CD64/CD206 expression. These findings indicate that PRP treatment effectively shifts SF-associated M1 macrophages to an M2-like phenotype, highlighting its potential as a therapeutic strategy for CHS.

## 1. Introduction

Repeated hemarthrosis in patients with hemophilia leads to chronic hemophilic synovitis (CHS), mainly because of iron accumulation, released from hemoglobin, in conjunction with its oxidized products in the synovia. This context triggers synovial hyperplasia, neoangiogenesis, secretion of inflammatory cytokines, the release of extracellular traps (ETs), and the recruitment of immune cells, including monocytes and neutrophils [1,2,3,4,5].

Monocytes are highly plastic circulating myeloid cells that differentiate into macrophages after extravasation into tissue. They are key effector cells not only in the initiation of an inflammatory response against an insult, but also in driving tissue repair, remodeling, and the maintenance of homeostasis. Macrophage functional diversity is explained by the ability to polarize into different phenotypes in response to microenvironmental conditions, being able to differentiate into a plethora of subtypes [6,7,8,9]. Thus, the local metabolites, growth factors, cytokines, and cell–cell interactions outline their phenotypes and functions. Moreover, during inflammation, recruited monocytes are able to repopulate tissue resident macrophages [10]. Definition and classification of macrophages are constantly updated, but they are frequently simplified into the pro-inflammatory type (referred as M1), and those with anti-inflammatory and tissue repair programs (M2), including subtypes such as M2a (IL-4/IL-13), M2b (TLR/IL-1R ligands), M2c (IL-10/glucocorticoids/TGF-β), and M2d (adenosine signaling/IL-6) [11]. Due to cellular plasticity, they constantly switch and transition between these states, possibly introducing intermediate phenotype populations with shared characteristics [12,13,14].

In CHS, iron overload, in the form of hemosiderin deposits, as well as IL-1α, IL-6, and TNF-α, can induce in macrophages a catabolic and pro-inflammatory program, resulting in an M1 phenotype [15,16]. These macrophages increase the production of nitric oxide and free radicals and release more inflammatory molecules, altering the metabolism of chondrocytes, and inducing apoptosis and upregulation of matrix metalloproteinases. These signals lead to cartilage extracellular matrix degradation, which affects joint function and contributes to the progression and establishment of the pathology [17,18]. Following an intra-articular bleed, the joint’s lymphatic system plays a crucial role in clearing blood components; however, recent studies have demonstrated that in the context of recurrent hemarthrosis (particularly in factor-deficient systems), this process becomes dysfunctional. This results in maladaptive or defective lymphangiogenesis, accumulating and delaying clearance of iron from the synovium. This retention of iron-laden macrophages is highly pathogenic for the tissue [19,20]. Current treatments in CHS are focused on reducing bleeding and synovectomy, without resolving the reduction in synovial inflammation and the regeneration of the joint to impair the progression of tissue damage [21,22].

Platelet-rich plasma (PRP), an autologous blood derivative obtained by centrifugation, has emerged as a novel therapeutic alternative in the field of regenerative medicine and for intra-articular treatment in several arthropathies, such as rheumatoid arthritis and osteoarthritis [23,24,25,26,27]. The therapeutic properties of PRP are based on the action of more than 300 pre-formed substances stored in platelet alpha granules, such as growth factors, chemokines, cytokines, and extracellular matrix modulators, among others. These molecules can regulate the immune system and induce cellular regenerative processes such as neoangiogenesis, connective tissue repair, monocyte migration, and the proliferation and differentiation of fibroblasts and mesenchymal stem cells in tissue-specific cells [28,29]. Furthermore, in vitro studies adding different formulations of PRP directly to macrophage cultures reduce co-stimulatory molecules (CD80 and CD86), and genes related to the NF-kB pathway (TNF-α, S100A12, and IL-6), or cytokines (TNF-α and IL-1β) [30,31,32,33].

In the context of CHS, different worldwide clinical groups, including our collaborative team, have reported that the intra-articular injection of PRP reduces bleeding episodes in patients with CHS and improves clinical parameters of articular damage [34,35,36,37,38]. In this sense, we have demonstrated that PRP dampens hemoglobin oxidation, as well as the release of ETs in vitro, as new and non-canonical mechanisms underlying the therapeutic effect of PRP in hemophilia [5,39]. Other preliminary in vitro evidence highlighted the potential role of macrophages in the clearance of ETs [40,41,42].

Here, we aimed to understand how synovial microenvironment conditions impact macrophage differentiation and polarization and how PRP modulates macrophage reprogramming to dampen the inflammatory response and promote tissue repair as a mechanistic therapeutic validation underlying PRP application in CHS patients.

## 2. Results

### 2.1. Hemosiderin Deposits and CD64+ Macrophages Characterize the Inflammatory Environment of SF of Patients with CHS

**Figure 1:**
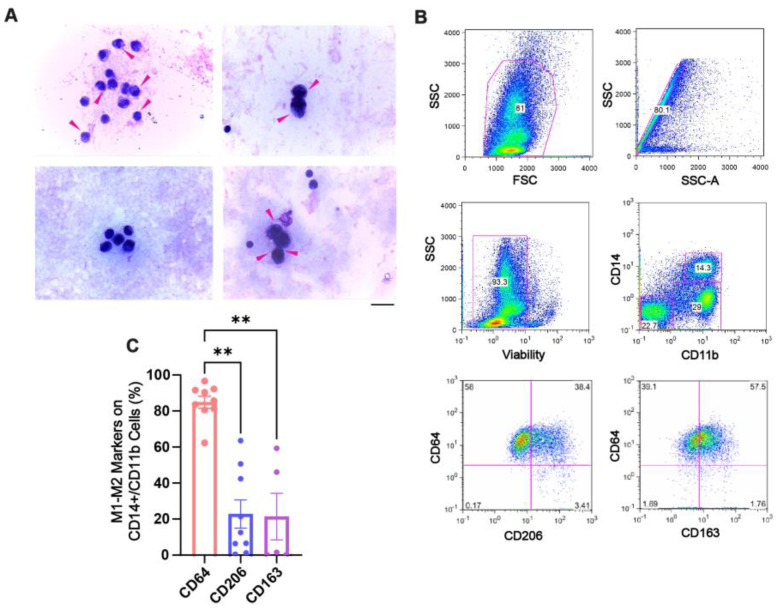
**Phenotypic characterization of SF macrophages from CHS patients before PRP treatment**. (**A**) Representative light microscopy images of Giemsa-stained SF samples from four patients (out of n = 22). Pink arrows indicate hemosiderin-laden macrophages. (Magnification: 400×; scale bar: 20 µm). (**B**) Flow cytometry gating strategy to identify SF macrophages (CD14^+^/CD11b^+^), which were subsequently analyzed for M1 (CD64) and M2 (CD206 and CD163) polarization markers. (**C**) Quantification of macrophage marker expression across patient samples. Data are presented as means ± SEMs (n = 5–9). ** *p* < 0.01, determined by Kruskal–Wallis test with Dunn’s multiple comparisons test.

To characterize macrophages from SF (synovial fluid) samples of CHS patients, we first stained them with Giemsa dye (microscopy) and then confirmed the immune phenotype using a validated directly conjugated panel of antibodies for macrophage polarization by flow cytometry [43,44]. Representative microscopy pictures of the SF smears showed synovial macrophages as large cells containing cytoplasmic hemosiderin deposits stained in dark violet–blue dots (Figure 1A, pink arrows). The presence of these loaded hemosiderin macrophages indicated a pro-inflammatory skewing that was confirmed by flow cytometry. After gating on live cells and the double-positive CD11b^+^/CD14^+^ macrophage population (Figure 1B), we observed a higher percentage of cells expressing CD64 (78.5 ± 4.2%), an M1 marker, compared with cells expressing CD206 (22.74 ± 8.4%) and CD163 (26.33 ± 15.4%), both classical M2 receptors (Figure 1C). Thus, synovial macrophages from CHS patients, previous to receiving PRP treatment, clearly showed a pro-inflammatory M1 profile. This was also supported by the presence of high levels of TNF-α, a prototypical inflammatory cytokine, and TGF-β, a pleiotropic cytokine considered a non-resolutive pro-fibrotic factor, in the SF from CHS patients compared with other arthropathies [45,46,47,48,49,50] (Supplementary Table S1).

### 2.2. The Induction of Pro-Inflammatory MDM by SF of Patients with CHS In Vitro

**Figure 2:**
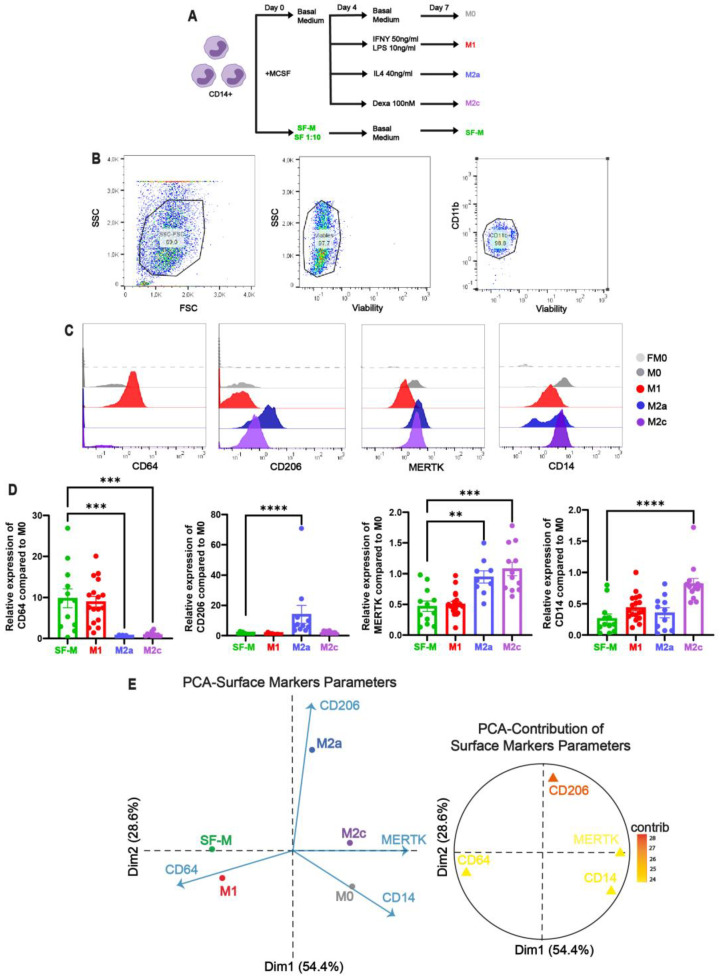
**Synovial fluid from CHS patients reprograms MDMs toward a pro-inflammatory phenotype in vitro.** (**A**) Schematic of the protocol used to differentiate MDMs in the presence of standard polarizing cytokines or patient-derived SF. (**B**) Representative flow cytometry gating strategy for identifying live CD11b^+^ macrophages after 7 days of culture. (**C**) Histograms showing the mean fluorescence intensity (MFI) for CD64, CD206, MERTK, and CD14 under each macrophage culture condition. (**D**) Relative MFI quantification for each marker and culture condition was compared with the M0 (non-polarized condition) and graphed. Significant statistical comparisons of SF-M against polarizing conditions (M1, M2a, and M2c) are indicated as ** *p* < 0.01, *** *p* < 0.001, and **** *p* < 0.0001 using Kruskal–Wallis test with Dunn’s multiple comparisons test (n = 9–19). (**E**) Plot of PCA surface marker expression analysis with the first two principal components (Dim1 and Dim2), showing the mean value of each condition and its association with the variables, together with their contribution graphs (n = 9–19).

To mimic the synovial context and deeply analyze macrophage reprogramming, we developed an in vitro differentiation model of monocyte-derived macrophages (MDMs) in the presence of SF from CHS patients (Figure 2A). We differentiated macrophages from sorted blood CD14^+^ monocytes of healthy donors in the presence of SF (1/10 *v*/*v*) added since day 0 (SF-Ms) and compared with non-polarized (M0) and polarized M1 (IFNγ/LPS), M2a (IL-4), and M2c (dexamethasone) macrophages by flow cytometry (Figure 2B–D).

These results clearly showed that SF from CHS patients exhibited significantly increased CD64 expression in SF-Ms, compared with M1. Similarly, SF-Ms showed significantly lower levels of CD206, MERTK, and CD14 compared with cardinally polarized M2a and M2c. To further validate and confirm our results, we performed a principal component analysis (PCA). This analysis allowed us to determine the relationship and weight among all surface markers together for each experimental condition (SF-M, M0, M1, M2a, and M2c). The cardinal points were the polarized macrophages based on the contributions of each variable to the total variation, and the SF-M dropped close to M1 in the bi-dimensional map of Dim1 (54.4%) and Dim2 (28.6%) (Figure 2E). This result demonstrated that the in vitro SF-Ms exhibited a skewing toward the M1 pattern, in agreement with the phenotype of macrophages observed in the SF of CHS patients.

### 2.3. The Gene Program Profile Induced in SF-M Is Distinctive from Other Polarized MDMs

**Figure 3:**
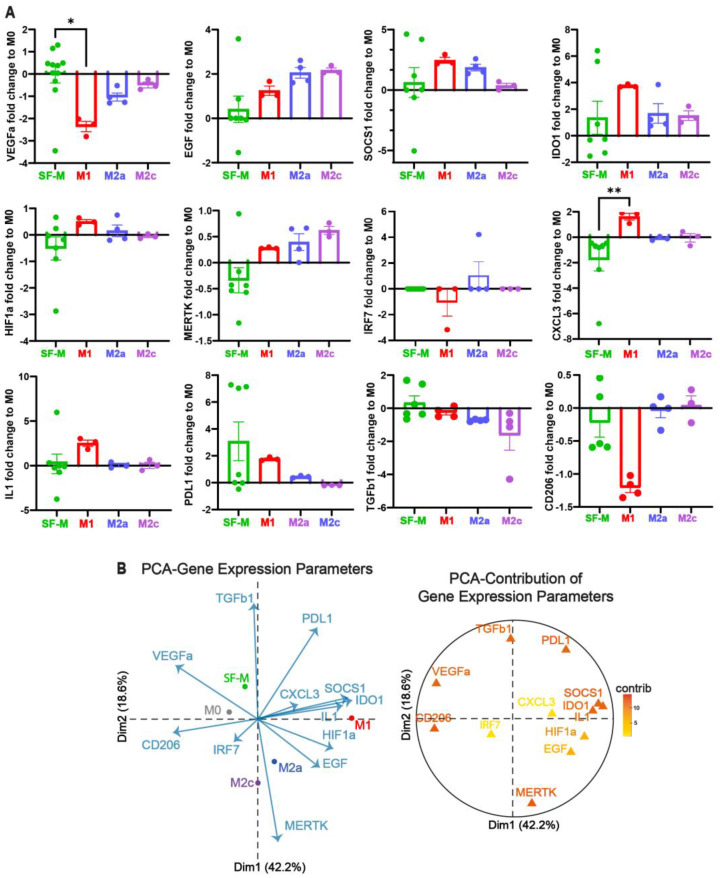
**The gene program profile induced in SF-Ms is distinctive from other cardinally polarized MDMs**. (**A**) Relative gene expression analysis comparing SF-M with M1, M2a, and M2c macrophages. Gene expression was quantified using the 2^−ΔΔCt^ method, normalized to the M0 (non-polarized) condition, and then log10-transformed. The housekeeping gene used was EF1A1. Each macrophage condition was distinguished by colors: SF-Ms versus the polarized macrophage conditions (M1, M2a, and M2c). Data are presented as means ± SEMs (n = 3–11). * *p* < 0.05 and ** *p* < 0.01, determined by one-way ANOVA or Kruskal–Wallis test with Dunn’s multiple comparisons test. (**B**) The PCA plot shows the clustering of each macrophage condition based on its transcript profile across the first two principal components (Dim1 and Dim2). An accompanying graph illustrates the contribution of individual genes to these components.

To characterize the gene expression re-programming induced in these SF-Ms compared with the polarized MDMs, we analyzed 12 selected genes (VEGFα, EGF, SOCS1, IDO1, HIF1α, MERTK, IRF7, CXCL3, IL1, PDL1, TGF-β, and CD206) based on our previous work [6,43] and the cross-tissue transcriptional landscape of human monocytes and macrophages in health and disease [51]. The individual relative gene expression profile, normalized to non-polarized M0, showed a distinctive gene pattern for the SF-Ms compared with the polarized macrophages characterized by differential expression of VEGFα, HIF1α, MERTK, CXCL3, and TGF-β, as shown in Figure 3A. To integrate all gene expressions for each MDM condition, we also generated a PCA map showing the first two main contributors (Dim1 and Dim2), as shown in Figure 3B. Here, SF-Ms clustered distantly from those differentiated with polarizing cytokines (M1, M2a, and M2c), mainly driven by the VEGFα, PDL1, and TGF-β vectors (Figure 3B), suggesting a specific gene program related to CHS. Taken together, our data suggest that SF from CHS patients promotes a unique MDM characterized by genes related to new vessel formation, hemarthrosis, and an unresolved inflammatory process.

### 2.4. Addition of PRP Is Able to Dampen the Inflammatory Signature of SF-Ms

**Figure 4:**
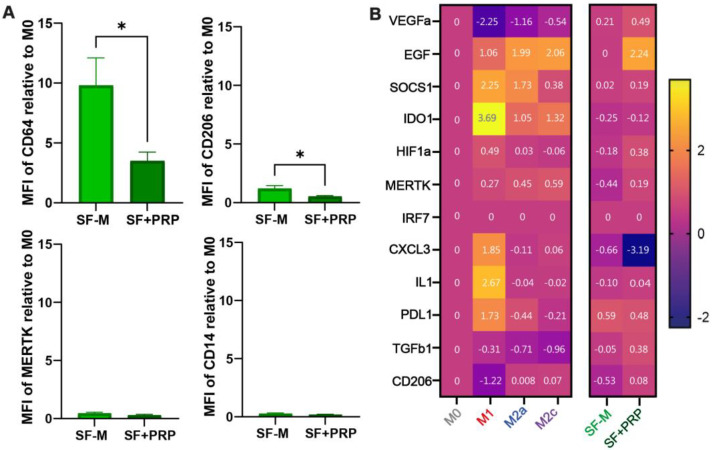
**PRP modified the inflammatory signature of monocyte-derived macrophages obtained in the presence of SF**. (**A**) Polarizing cell surface markers in SF-Ms were analyzed after PRP treatment (10% *v*/*v*), and data are referred to as fold change of MFI to M0; * *p* < 0.05; Mann–Whitney test; n = 6–12. (**B**) A heat map of transcript expression was generated by the 2^−ΔΔCt^ method, normalized to M0, and transformed by the log10 function. The housekeeping gene used was EF1A1. Numbers inside colored squares are the relative median gene expression values for a given condition; n = 3–11.

First, we showed that PRP is able to directly promote an M2 phenotype when added to monocyte-derived differentiated macrophages from healthy donors on day 4 (Supplementary Figure S1), as previously reported using different hemoderivative formulations [30,31,32,33]. However, here, we aimed to evaluate whether PRP is able to reprogram the already skewed inflammatory SF-M to a tissue repair MDM. We added PRP from healthy donors (10% *v*/*v*) on day 4 and analyzed the phenotype and transcriptional reprogramming of SF-Ms. As shown in Figure 4A, the 10-fold induction of CD64+ cells (M1 marker) was significantly reduced (* *p* < 0.05) after adding PRP (SF + PRP). Moreover, the addition of PRP also further reduced the CD206 receptor in these macrophages. No significant changes were observed in MERTK or CD14 expression with the addition of PRP to the SF-Ms (Figure 4A). Analyzing cytokine levels in the macrophage’s supernatant by ELISA, no differences were observed in IL-6, TNF-α, or IL-10 comparing SF-M vs. SF-M + PRP conditions (Supplementary Figure S2). Surprisingly, the addition of PRP to the SF-Ms significantly increased the relative expression of TGF-β (Supplementary Figure S2). We then analyzed the effect of PRP addition on the expression of 12 genes, comparing the SF-Ms vs. SF + PRP, as shown in Figure 4B. The heat map shows that PRP addition mainly increased the EGF level but also showed some changes in SOCS1, VEGF, HIF1a, TGFb1, and MERTK. An important reduction in CXCL3 was also observed. Even if the differential gene expression did not reach statistical significance, taking together the modulatory effect on surface markers and induction of TGF-β, these results further confirm that PRP treatment modulated the SF-induced profile.

Finally, we used a random forest (RF) machine learning algorithm with public scRNA-seq data and the MoMacverse framework [51] to compare M1 versus M2 transcript divergence. This allowed us to test the expression patterns of our 12 target genes. The model, trained on datasets detailed in the Materials and Methods Section, demonstrated a good performance on a test dataset (N = 23), reaching accuracy (Ex) = 91.3%, sensitivity (S) = 60%, specificity (Es) = 100%, kappa (K) = 0.7, and area under the curve (AUC) = 0.8. This constitutes an acceptable model for classifying cells into M1 or M2 strains based on the count values of the 10 selected target genes. All (100%) of the M1 cells were correctly identified, while 60% of the M2 cells were correctly classified. This discrepancy could be attributed to the number of cells used to train the model, which favored M1 by almost 4 to 1. A ranking of genes was extracted based on the mean decrease in the Gini index (Supplementary Figure S3A), identifying HIF1A and IL1A as better contributing genes for the separation groups. The resulting model was then tested in two experiments to predict the proportion of M1 vs. M2, comparing SF-Ms vs. SF + PRP. The model predicted that both SF-Ms (N1 and N2) were predominantly M1 (75.6% and 71.4%, respectively), which were reduced with PRP (SF + PRP) in both cases, to 60.2% and 56.6%, respectively (Supplementary Figure S3B).

### 2.5. Cellular Reconstitution of and Reduction in CD64 Macrophage After PRP Treatment in the SF of CHS Patients

**Figure 5:**
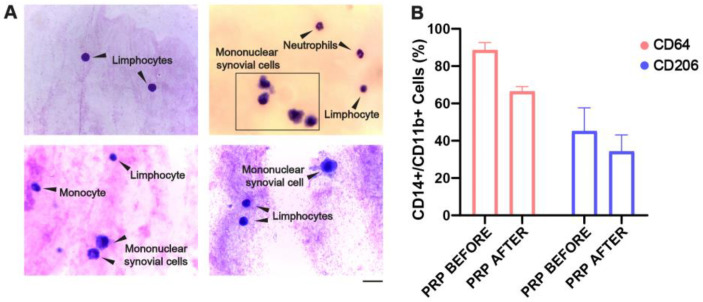
**PRP treatment promotes cellular reconstitution of synovia and dampens pro-inflammatory macrophages in CHS patients**. (**A**) Representative images of Giemsa-stained smears of synovial fluid samples from CHS patients after 2 weeks of PRP treatment (magnification: 100X; scale bar: 20 μm; n = 5). (**B**) Percentage of CD14+/CD11b+ cells expressing CD64 and CD206 in SF of patients after 2 weeks of PRP treatment (n = 3) evaluated by flow cytometry.

Interestingly, we were able to evaluate the SF composition after 2 weeks of receiving an intra-articular injection of PRP in five CHS patients (n = 5) using Giemsa staining and light microscopy analysis. As shown in Figure 5A, the Giemsa-stained SF smears exhibited the absence of macrophage-loaded or hemosiderin deposits with normal cellular content of monocytes, synovial cells, and lymphocytes. Furthermore, flow cytometry analysis in three follow-up patients showed a decreasing trend in CD11b^+^CD14^+^CD64^+^ macrophages (Figure 5B). Similar to the in vitro results, the addition of PRP also reduced the proportion of the CD206^+^ cell population. Due to the small sample size, no significant statistical difference was reported. Finally, we also found that the synovial levels of TNF-α and TGF-β were reduced after PRP treatment (Supplementary Table S2), as measured by ELISA. In contrast, we observed a slight increase in IL-6 levels after PRP treatment, highlighting the dual role of IL-6 signaling, which may also promote tissue regeneration, and no changes were observed in IL-10 levels (Supplementary Table S2).

### 2.6. SF-Ms Treated with PRP Generate Multinuclear Giant Cells When Challenged with NETs

**Figure 6:**
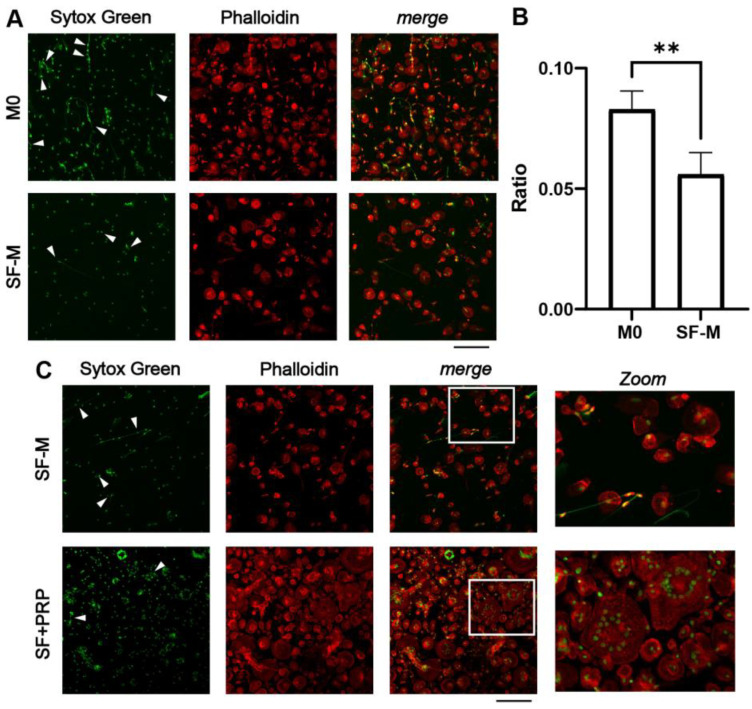
**Decreased NET clearance by SF-Ms and multinucleated giant cell formation with PRP treatment.** (**A**) NETs were generated from neutrophils of healthy donors, induced with 10% SF from CHS patients for 180 min, and stained with Sytox Green (white arrows). These NETs were added on day 7 of culture for 60 min to macrophages differentiated with basal medium (M0) or 10% SF. After co-culture, the samples were washed, fixed, and stained with phalloidin (phalloidin panel). (**B**) The phagocytosis ratio was calculated based on the number of cells displaying a Sytox Green-positive signal within the cytosol, relative to the total number of cells per image. ** *p* < 0.01; Mann–Whitney test (n = 6). (**C**) Macrophages were differentiated as already described, by adding PRP on day 4 of differentiation (SF + PRP). Pre-stained NETs with Sytox Green were added to 7-day macrophage cultures and incubated for 60 min. The multinucleated giant cells’ formation was considered when more than 3 nuclei were observed. Images were acquired by fluorescence confocal microscopy (20×; scale bar: 20 µm). The merged panel shows co-localizing cells with NETs, accompanied by magnified insets (outlined in white) (n = 6).

How macrophages contribute to the elimination of ETs in the context of CHS has not been investigated. Here, we aimed to understand how the ETs from neutrophils or NETs are processed by MDMs differentiated with SF (SF-M) or SF + PRP. We first found that phagocytosis of SF-induced NETs (pre-labeled with Sytox Green) by 7-day MDMs occurred mainly after 60 min of co-culture (Supplementary Figure S4). We then demonstrated that the SF-Ms exhibited a significantly lower proportion of cells with cytoplasmic NETs signals compared with the basal (M0) condition, evaluated by confocal microscopy (Figure 6A,B), as expected for M1-like macrophages. When we used a 7-day SF + PRP-treated macrophage condition for the phagocytosis assay, we consistently observed the rapid emergence of large, multinucleated giant cells in a notable proportion of the culture, as shown in the representative confocal microscopy image (Figure 6C). This assay was repeated multiple times with the same result, confirming that the appearance of these multinucleated giant cells mainly occurred when macrophages, differentiated in the presence of SF and PRP (SF + PRP), were exposed to NETs. This phenomenon may result from macrophage fusion, considering the short time elapsed following NETs’ addition. Supporting this hypothesis, and in concordance with the M2 reprogramming of SF + PRP, previous studies have shown that M2-polarized macrophages are more prone to cell fusion in the presence of foreign bodies [42,52,53]. Nonetheless, adding IL-4 to SF-Ms was not enough to recapitulate giant cell formation when co-cultured with NETs (Supplementary Figure S5). While the formation of these giant cells prevented accurate quantification of phagocytosis under these conditions, it opens a new avenue for exploring alternative strategies for the removal of large structures such as NETs.

## 3. Discussion

Intra-articular bleeding, or hemarthrosis, is one of the most severe manifestations in patients with hemophilia, leading to the development of chronic hemophilic synovitis, with no current available treatment promoting tissue regeneration [1,21]. In this sense, several clinical studies have demonstrated the therapeutic potential of intra-articular PRP injections, showing improvements in joint health, as measured by the Hemophilia Joint Health Score (HJHS), the frequency of bleeding episodes, the Visual Analog Scale (VAS), or pain scores, among others [34,35,36,37,38,54]. However, the mechanisms underlying PRP’s beneficial action in CHS patients remain largely unknown. While many publications support its use, controversy persists regarding its efficacy. A systematic revision in 2018 indicated that there is weak evidence, and high-quality comparative trials are needed [55]. A recent large meta-analysis (up to 2024, including 11 randomized controlled studies) found that while pain relief (VAS scores) favored PRP over a placebo at 3 and 6 months, this benefit was lost at 12 months [56], noting significant heterogeneity. Regarding painful hemophilic arthropathy, the use of PRP injections has also been discussed [54,57]. The number of injections needed for long-term efficacy and the endpoint of studies are also not well defined, and further investigation is needed to evaluate the cost of such treatment.

Our work aimed to contribute to the understanding of how PRP’s modulatory effects, through macrophage reprogramming, dampen inflammation and induce tissue repair, ultimately promoting joint recovery in CHS patients.

We first characterized the phenotype of ex vivo macrophages isolated from the synovial fluid (SF) samples of CHS patients before PRP treatment. These macrophages showed abundant intracellular hemosiderin accumulation, along with a high proportion of cells expressing the FcγRI (CD64) receptor, and a low proportion expressing the M2-associated mannose scavenger (CD206) and hemoglobin–haptoglobin scavenger (CD163) receptors, demonstrating a predominant pro-inflammatory M1 environment within the synovial. Previous studies have also shown the presence of iron deposits or iron derivatives in the synovial tissue of hemophilic patients as an inflammatory marker, underscoring iron’s contribution to disease chronicity, and distinguishing CHS from other joint diseases, such as rheumatoid arthritis (RA) and osteoarthritis (OA) [15,58,59,60]. Our study is the first to define the surface cell phenotype of CHS synovial fluid macrophages, with a clear inflammatory M1 shift. Furthermore, we validated that the synovial fluid microenvironment of CHS joints was able to phenocopy inflammatory macrophages (SF-Ms) in vitro under SF stimulation. This model recapitulated the ex vivo observations and gave us the opportunity to test the impact of PRP on the polarization and reprogramming of SF-Ms.

Most previous studies have focused on iron’s role as a macrophage-polarizing factor [15,61,62] and have largely ignored other bioactive components present in synovial fluid, including cytokines, plasma proteins, extracellular traps (ETs), and cellular debris, which may significantly influence macrophage polarization [22,63].

Our results demonstrate that SF-Ms not only acquired an M1 inflammatory phenotype but were also characterized by a unique transcriptional profile, with the upregulation of VEGFα and TGF-β1, distinctive from classical M1 polarization driven by IFNγ plus LPS. Both VEGFα and TGF-β1 may contribute to neovascularization, as previously reported in CHS patients [64,65], with synovial macrophages potentially acting as a source of VEGFα. Additionally, persistent TGF-β1 signaling could sustain chronic inflammation and fibrosis-like responses. M1 polarization of macrophages in the synovial environment has also been documented in other arthropathies, such as OA and RA, and has been associated with disease severity [66,67,68,69].

Our findings demonstrate that the M1-like profile of these macrophages can be modulated by PRP treatment, both in vitro and ex vivo. We observed a reduction in surface marker expression, particularly CD64, as well as increased TGF-β levels in culture supernatants and activation of a transcriptional program consistent with tissue repair signaling (including increased levels of EGF, MERTK, and TGF-β, alongside a marked decrease in CXCL3 levels). TGF-β has been described as a key regulator with a broad range of effector functions, including M2 macrophage polarization [70,71] and modulation of different immune cells [30,31,72]. M2 tissue repair signaling involves activation of the PIK3/AKT, STAT3/6, and SMAD2/4 pathways, coupled with a reduction in mTOR and NF-κB signaling [30,73,74], which is concordant with our gene response after PRP reprogramming of SF-M.

Additionally, the beneficial effects of PRP are mediated by a complex cocktail of growth factors, cytokines, and, significantly, extracellular vesicles like exosomes, which can also impact lymphatic function by promoting lymphatic vessel integrity and drainage. Recent research demonstrates that exosomes derived from PRP can transfer bioactive molecules (like microRNAs or proteins) that stimulate the proliferation, migration, or survival of lymphatic endothelial cells and promote regeneration and repair [75,76].

Once we established that PRP skewed inflammatory SF-Ms toward a tissue repair subtype, we assessed macrophage functionality by examining ETs’ clearance. These damage-associated molecular pattern (DAMP) structures are abundantly present in the synovial fluid of CHS patients and animal models, and they are associated with joint damage [5,77]. Thus, their clearance may contribute to the therapeutic benefits of PRP. Consistent with pro-inflammatory M1 macrophages, decreased phagocytosis of ETs was observed in SF-Ms. However, the exposure of SF-Ms + PRP to ETs surprisingly showed the formation of multinucleated giant cells. This result was very interesting since, considering the short time of co-culture interaction, these giant macrophages can most probably come from the fusion of cells, facilitating the clearance of undigested ET structures. Multinucleated giant cells have been observed in chronic inflammation, both in infectious contexts (e.g., granuloma formation in tuberculosis) and in response to biomaterial implants. In the latter, when particles exceed the phagocytic capacity of single macrophages (10–100 μm), foreign body-type giant cells are formed. These cells have been reproduced in vitro via IL-4 or IL-13 stimulation, cytokines that also promote M2 macrophage polarization [78,79]. This suggests that SF-M + PRP-treated M2-like macrophages may have greater potential to form such structures compared with cells exposed only to CHS synovial fluid.

The mechanisms by which macrophages phagocytize ETs remain under debate; however, some preliminary evidence from in vitro and ex vivo studies has indicated their potential role in the clearance of ETs [40,41,42]. Recent findings show the presence of phosphatidylserine on ETs, suggesting a role for phospholipid-recognizing receptors or opsonization by the complement component C1q [80,81,82]. Even though phagocytosis could explain some clearance of ETs, particularly those that were already DNAse-digested, the bigger size of undigested ETs or those still coupled with cells raises the possibility of multinucleated giant cell formation as a response to big structures [40,42]. Ultimately, defining the specific macrophage responses to ET stimuli in the CHS environment—and elucidating mechanisms for ET clearance—are key issues that remain to be addressed. The induction of macrophage fusion and giant cell formation in response to synovial induced-ETs, promoted by PRP treatment, may represent a promising mechanism for ET removal and further support PRP’s therapeutic potential.

## 4. Materials and Methods

### 4.1. Ethical Considerations

This study was performed according to the ethical and legal requirements established in the national ANMAT6677/10 and international bioethical regulations Nuremberg Code, the Declaration of Helsinki, and the Universal Declaration on Human Genome and Human Rights of the General Conference of UNESCO, 11 November 1997. The recruitment of patients with CHS, sample acquisition, healthy donors’ blood sampling, and in vitro experiment design were approved by the Ethics Committees of Hospital Fernández and the National Academy of Medicine.

### 4.2. Patients, Treatment, and Obtention of Synovial Fluid (SF)

A total of 22 patients were included in the protocol, and 23 joints (1 ankle and 22 knees, where 1 was bilaterally affected) were treated at the Hospital Fernández and Hemophilia Foundation (Buenos Aires, Argentina) between July 2018 and January 2020. Twenty patients were hemophilia type A [eighteen were severe (including one with an inhibitor), and two were moderate], and two were type B (severe). The mean age was 28 years (13–60 years old). The patient demographics are shown in Table 1. The inclusion criterias were patients with chronic synovitis diagnosed by a clinical exam and ultrasound, and confirmed by magnetic resonance images. Those with hemophilic arthropathy grade 5, according to the radiological classification of Arnold and Hilgartner (bony ankylosis), as well as a clinical evaluation (exhibiting limited intra-articular injection, such as compromised skin integrity, altered venous access, or active joint infection), were excluded from this study.

All patients received one intra-articular injection of PRP as routine treatment performed by Dr Caviglia’s clinical group at the Hospital Fernández and Hemophilia Foundation, as previously reported [5,34,39]. To prepare the PRP, briefly, 15 to 25 mL of blood was collected (BD Vacutainer, 8.5 mL ACD tubes) and centrifuged at 360× *g* for 8 min at room temperature. This yielded 4 to 7 mL of PRP, which was carefully collected to avoid erythrocyte and leukocyte contamination. Prior to injection of PRP, the synovial fluid (SF) was aspirated from the joint using a 21G needle, and an equivalent volume of PRP (a maximum of 5 mL) was then injected to match the amount of SF removed. Twenty-three SF samples were obtained from twenty-two patients pre-PRP injections. These samples were used for cellular analysis via microscopy and macrophage phenotype characterization via flow cytometry. We also obtained SF samples from a small cohort of 5 patients after 2 weeks of follow-up from the application of one intra-articular injection of PRP.

### 4.3. Synovial Fluid (SF) Processing

Aspirated fresh SF samples (3–10 mL), before or after PRP treatment, were used for laboratory analysis and ex vivo characterization, as follows. A drop of a fresh SF sample was spread onto a glass slide and stained with Giemsa, and the cellular content was examined by light microscopy. The remainder of the SF was centrifuged at 350× *g* for 10 min to separate the acellular supernatant from the cellular pellet. The acellular supernatant was carefully collected, aliquoted, and stored at −20 °C for later use. The cellular pellet was resuspended in phosphate-buffered saline (PBS), and residual red blood cells were lysed by incubating the suspension in an ammonium chloride-based buffer for 5 min. The resulting cell suspension was then analyzed for macrophage phenotyping using flow cytometry, as described below.

### 4.4. Platelet-Rich Plasma (PRP) Isolation for In Vitro Assays

PRP from healthy donors’ blood samples was obtained by venous puncture from individuals who had not taken non-steroidal anti-inflammatory drugs during the 10 days before sampling. None of the healthy donors has any coagulopathy. Blood was collected in tubes containing 3.8% trisodium citrate (1:9 *v*/*v*) and centrifuged at 180× *g* for 10 min. PRP was collected, avoiding contamination with red and white blood cells. Then, coagulation was induced by adding CaCl_2_ (22–25 mM) at 37 °C for 40 min. The clot was removed, and the PRP supernatant was centrifuged at 890× *g* for 10 min and then stored at −80 °C until use.

### 4.5. Peripheral Blood Mononuclear Cell (PBMC) Isolation

The PBMCs were isolated from the same peripheral blood of healthy volunteers after PRP was removed (N = 28; median age: 30 years), following a standardized protocol as we previously described [6,83]. Briefly, the cellular blood fraction, after PRP separation, was half-diluted (*v*/*v*) in PBS and used to obtain the PBMCs by density gradient centrifugation (400× *g* for 30 min at room temperature and without brakes) employing Ficoll-Paque Plus (1078 g/mL density, GE Healthcare, Marlborough, MA, USA). The PBMCs’ recovered fraction was washed twice with PBS plus 2% FBS (fetal bovine serum).

### 4.6. CD14 Purification and In Vitro Monocyte-Derived Macrophage (MDM) Differentiation

CD14+ monocytes were isolated from PBMCs using the EasySep Human CD14 positive selection kit (STEMCELL Technologies, Vancouver, Canada), following the manufacturer’s instructions, as we previously reported [6,83]. Assays were performed by seeding 2 × 10^5^ isolated CD14+ cells in 48-well plates and culturing in complete RPMI-1640 medium (10% FBS, 1% penicillin–streptomycin, and 50 ng/mL of M-CSF) at 37 °C and 5% CO2. Monocytes were differentiated into MDMs for seven days in the presence of SF (1/10 dilution) from day 0 (SF-M), with or without the addition of PRP (1/10 dilution) on day 4 (SF + PRP), and left for 72 additional hours for analysis. As controls and standardized profiles, non-polarized (M0) and polarized cells with IFNγ (50 ng/mL) plus LPS (10 ng/mL) for M1, IL4 (40 ng/mL) for M2a, and dexamethasone (100 nM) for M2c were run, as previously reported [43,44].

### 4.7. Neutrophil Isolation and Purification

After PBMCs’ isolation, the remaining blood containing the red blood cell pack and polymorphonuclear cells was mixed with a 6% dextran solution (Sigma Aldrich, St. Louis, MO, USA). After erythrocyte sedimentation, neutrophils were collected from the upper phase, where the remaining red blood cells were lysed with distilled water, and the resulting neutrophils were resuspended in RPMI-1640 medium (GE Healthcare, Buckinghamshire, UK) supplemented with 1% FBS. The purity of the cell suspensions and the percentage of live/apoptotic/necrotic cells were determined by microscopy by staining with acridine orange and ethidium bromide, respectively. The purity and viability of neutrophils used in the experiments in this study always exceeded 98%.

### 4.8. Surface Staining and Flow Cytometry

To analyze the polarization skewing, we used a previously validated macrophage surface staining panel [43,44,83], employing the appropriate combination of conjugated antibodies (BioLegend) against human CD11b-APC/Cy7 or BV421 (RRID: AB_830641), CD64-APC (RRID: AB_1595539), CD163-PerCP/Cy5.5 (RRID: AB_2650629), CD206-AlexaFluor 488 or FITC (RRID: AB_571874), CD14-PECy7 (RRID: AB_830691), and MERTK-biotin (RRID: AB_2721500), together with a streptavidin–PE (BioLegend, San Diego, CA, USA), and following the standardized laboratory protocol. Briefly, the harvested cells were washed with PBS and blocked with PBS/5% FBS at room temperature for 15 min. The cells were washed with PBS, and the respective antibody panel (prepared in PBS/2% FBS) was added to the cell pellet and incubated at 4 °C for 30 min. A fixable viability dye, Zombie Violet or NIR (both from BioLegend), was used according to the manufacturer’s instructions to gate on live cells. After washing, the cells were fixed with a Cytofix/Cytoperm Kit (BD Biosciences, San Jose, CA, USA) and washed again. The cells were acquired using a FACS Canto I cytometer (Becton Dickinson, San Diego, CA, USA) or a Sysmex CyFlow Partec (Görlitz, Germany), and all the analysis was carried out with the FlowJo software 8.2 (Tree Star). The negative control fluorescence minus one (FMO) was used to set a negative signal in the interested channels.

### 4.9. ELISA

SF from CHS patients and supernatants from MDM cultures were collected and stored at −80 °C until cytokine measurement. Any cellular debris was removed by centrifugation at 2100× *g* for 5 min. Commercial ELISA kits for IL-6, TNF-α, IL-10 (Biolegend), and TGF-β (R&D) were used, following the manufacturer’s instructions, and the absorbance at 450/570 nm was determined using a plate reader (OASYS, UVM 340, London, UK).

### 4.10. Quantitative PCR (qPCR)

For gene expression analysis, the culture medium of MDMs was discarded and washed with PBS, and then harvested with BioZol (PB-L Productos Bio-Lógicos, Quilmes, Argentina), following the manufacturer’s instructions. Reverse transcription was performed using 300–500 ng of RNA in 20 μL of the reaction volume by employing an iScript cDNA synthesis kit (Bio-Rad, Hercules, CA, USA). Real-time qPCR reactions were assessed using 1 μL of cDNA in 10 μL of the reaction volume by employing SsoAdvanced universal SYBR Green mix and CFX-Connect equipment (Bio-Rad, Hercules, CA, USA). The primers used in this study are listed in Table 2. The reaction was normalized to housekeeping gene expression levels and the elongation factor EEF-1 alpha 1 (EEF1A1), and the specificity of the amplified products was checked through analysis of dissociation curves. To generate the graphics (the PCA or the heat map), the relative expression obtained by the 2^−ΔCt^ method was normalized to reference values (non-polarized or M0) and transformed by the log10 function to scale up intergenic expression differences and individual variations. In the case of the heat map, numbers inside colored squares were the mean value from each group in a given condition.

### 4.11. Comparison of SF-Ms and SF + PRP Gene Profiles in the MoMacverse Framework

Processed scRNAseq data tables were obtained from the GEO database (GSE178209). In the study in [51], various clusters, referred to as phenographs, were identified, which were associated to varying degrees with the M1 and M2 macrophage strains. This study obtained the M1-exclusive phenograph (phenograph 4; N = 349) and the M2-exclusive phenographs (phenographs 2, 16, and 17; N = 94). After filtering the data to retain only the cells representative of these phenographs, we further filtered the dataset to focus on the 12 genes under study: “CXCL3”, “EGF”, “HIF1A”, “IDO1”, “IL1A”, “IRF7”, “MERTK”, “PDL1”, “SOCS1”, “VEGFA”, “TGFB1”, and “CD206”. The resulting dataset, composed of 443 cases (349 + 94) and 12 variables (12 genes), was randomly divided in a balanced manner, with 95% (N = 418) used for training the RF models and 5% (N = 23) for testing. The 2^−ΔCt^ values from q-PCR were transformed into pseudo-counts by normalization against the M0 internal control value for each experiment (N = 4). RNAseq count values and qPCR pseudo-counts were transformed using a regularized logarithm with the DESeq2 package in RStudio. In the training group, RF models were trained and optimized (mtry) using repeated cross-validation (k = 5; R = 15) with the caret package in RStudio. The resulting model was tested with the test dataset, and the following performance metrics were recorded: accuracy (Ex), sensitivity (S), specificity (Es), kappa (K), and area under the curve (AUC). Subsequently, the model was applied to the qPCR data, and the prediction results were provided not only as the final M1 or M2 classification but also in terms of the probabilities for each strain (M1 and M2).

### 4.12. In Vitro ET Formation Induced by Synovial Fluid from Patients with CHS

ETs were generated from neutrophils of healthy donors in the presence of 10% SF from CHS patients, incubated for 180 min at 37 °C with 5% CO_2_, as previously described [5]. After the incubation period, neutrophil extracellular traps (NETs) were collected by vigorous pipetting and stained with Sytox Green (1:1000) (Thermo Fisher, Waltham, MA, USA) for 15 min at room temperature, protected from light. These labeled NETs were used for macrophage co-cultures.

### 4.13. Co-Culture of NETs with MDMs, SF-Ms, and SF + PRP–Macrophages to Analyze ET Clearance

Macrophages used in these assays were differentiated over 7 days in the presence of basal medium (M0), with 10% SF from day 0, and supplemented or not with 10% PRP on day 4 of culture. Macrophages (300,000–400,000 cells/well) were differentiated in 24-well plates containing 12 mm glass coverslips coated with poly-D-lysine (50 µg/mL) (Thermo Fisher) to allow analysis by confocal microscopy. For the condition supplemented with PRP, excess PRP was removed by washing before the addition of NETs to avoid effects independent of macrophage action on NETs. The macrophage-to-neutrophil ratio tested was 1:5 or 1:10. To analyze NETs’ phagocytosis or interaction with macrophages, we used an adapted protocol from Lazzaretto et al. [84] by co-culturing for 1 h and then analyzing by confocal fluorescence microscopy.

After the co-culture period, the medium was removed, and the cells were fixed with 4% PFA for 15 min and stained with TRITC–phalloidin (1:1000) (Sigma). Coverslips were mounted with Polymount^®^, and images were acquired using an FV-1000 confocal fluorescence microscope (Olympus, Tokyo, Japan). The images were analyzed using the ImageJ software v. 1.54k, where the proportion or ratio of macrophages phagocytosing NETs was calculated based on the number of cells showing Sytox Green staining within their cytoplasm, relative to the total cell count per field.

### 4.14. Statistical Analysis

The data are each expressed as the mean ± standard error of the mean (SEM). The Shapiro–Wilk test was used to define state normality and equal variance. For comparisons between two groups, an unpaired *t*-test or the Mann–Whitney U test was used accordingly. For comparisons involving more than two groups, a one-way analysis of variance (ANOVA) or a Kruskal–Wallis test with Dunn’s multiple comparisons test was used, as appropriate. All statistical descriptions used in each experiment are described in their respective figure legends. A minimum of 3 and a maximum of 26 independent samples were employed, depending on the experiment run. Statistical significance was set at *p* < 0.05. The analysis was performed using the GraphPad Prism software 9.0.

To identify hidden patterns in the dataset, reduce its dimensionality by removing the noise and redundancy, and identify correlated variables, principal component analysis (PCA) was computed in RStudio 1.4.1106 using the missMDA, FactoMineR, FactorExtra, GridExtra, Plotly Ggplot2, and Corrplot packages. First, NA missing values were imputed, and continuous variables were detected automatically. Eigenvalues were then visualized, alongside the percentage of variance explained by each principal component. Graphics and clustering analysis were performed using the FactorExtra, Ggplot2, Plotly, and Corrplot packages.

## 5. Conclusions

In conclusion, our findings demonstrate that the synovial microenvironment in CHS patients is capable of differentiating and polarizing macrophages toward a pro-inflammatory M1-like phenotype, characterized by a gene expression program that appears distinctive to its pathological context. The shift from an M1 to a resolving M2 phenotype induced by PRP treatment suggests that macrophage reprogramming may underlie the therapeutic effects of intra-articular PRP injections in CHS. Further studies, particularly longitudinal ones, are needed to identify the specific molecules involved in M1 polarization, as well as to elucidate the mechanisms driving M2 macrophage modulation in response to intra-articular PRP therapy.

### Limitations of the Study

A significant limitation inherent to PRP research is the wide variability in the preparation of PRP itself, including several key factors: platelet concentration, activation method, leukocyte content, dosing, and volume. Another limitation was the relatively small sample size of our study. Another critical limitation of this study was the small clinical sample size for some specific assays. Future work with larger patient cohorts is needed to strengthen these results. Finally, our findings indicate that synovial fluid from CHS patients polarizes monocyte-derived macrophages toward an M1 profile and that PRP can shift this polarization to an M2 phenotype. However, the specific molecular pathways and transcriptomic reprogramming driving this shift have not been fully elucidated.

## Figures and Tables

**Table 1:** Patient demographic data at admission.

Variable	Patients	Joints
N (Male)	22	23
Age in years	28 (13:60)	
Severity and type of hemophilia	20 severe and type A; 2 moderate and type B	
Joint		22 knees; 1 ankle

**Table 2:** Human primers list.

Gene Name	5′-3′ Forward Sequence	5′-3′ Reverse Sequence
*Eef1A1*	TCGGGCAAGTCCACCACTAC	CCAAGACCCAGGCATACTTG
*VEGF*	ATGAGGACACCGGCTCTGACCA	AGGCTCCTGAATCTTCCAGGCA
*EGF*	CTTGGGAGCCTGAGCAGAAA	TGCACAAGTGTGACTGGAGG
*SOCS1*	CACGCACTTCCGCACATTC	TAAGGGCGAAAAAGCAGTTCC
*IDO1*	GATGTCCGTAAGGTCTTGCC	TCCAGTCTCCATCACGAAAT
*HIF1α*	ACTAGCCGAGGAAGAACTATGAA	TACCCACACTGAGGTTGGTTA
*MERTK*	CTCTGGCGTAGAGCTATCACT	AGGCTGGGTTGGTGAAAACA
*IRF7*	CAGCGAGTGCTGTTTGGAGAC	AAGTTCGTACACCTTATGCGG
*CXCL3*	CGCCCAAACCGAAGTCATAG	GCTCCCCTTGTTCAGTATCTTTT
*IL1*	CTGAACTGCACGCTCCGGG	GCTTATCATCTTTCAACACGCAGG
*PDL1* (CD274)	GCTTTTCAATGTGACCAGCA	GATGGCTCCCAGAATTACCA
*TGFb1*	GGAAATTGAGGGCTTTCGCC	CCGGTAGTGAACCCGTTGAT
*MRC1* (CD206)	AGCCAACACCAGCTCCTCAAGA	CAAAACGCTCGCGCATTGTCCA

*Eef1A1* (eukaryotic translation elongation factor 1 alpha 1); *VEGF* (vascular endothelial growth factor); *EGF* (endothelial growth factor); *SOCS1* (suppressor of cytokine signaling 1); *IDO1* (indoleamine 2,3-dioxygenase 1); *HIF1a* (hypoxia-inducible factor 1 subunit alpha); *MERTK* (MER tyrosine kinase); *IRF7* (interferon regulatory factor 7); *CXCL3* (C-X-C motif chemokine 3); *IL1* (interleukin 1); *PDL1* (programmed death-ligand 1, CD274); *TGFb1* (tissue growth factor beta 1); *MRC1* (mannose receptor C-type 1, CD206).

## Data Availability

The original contributions presented in this study are included in the article/Supplementary Material. Further inquiries can be directed to the corresponding author.

## Supplementary Materials

The following supporting information can be downloaded at https://www.mdpi.com/article/10.3390/ijms262110616/s1.

## Abbreviations

The following abbreviations were used in this manuscript:

CHS	Chronic Hemophilic Synovitis
ETs	Extracellular Traps
FBS	Fetal Bovine Serum
FMO	Fluorescence Minus One
HJHS	Hemophilia Joint Health Score
MDM	Monocyte-Derived Macrophage
NETs	Neutrophil Extracellular Traps
OA	Osteoarthritis
PBS	Phosphate-Buffered Saline
PBMC	Peripheral Blood Mononuclear Cell
PCA	Principal Component Analysis
PRP	Platelet-Rich Plasma
RA	Rheumatoid Arthritis
SF	Synovial Fluid
VAS	Visual Analog Scale
