# Supplementary material for: Platelet Releasate Reprograms Synovial Macrophages In Vitro: A New Approach in the Treatment of Hemophilic Synovitis
Source: Int J Mol Sci. 2025 Oct 31;26(21):10616. doi: 10.3390/ijms262110616 (PMC12610548; doi:10.3390/ijms262110616)
Supplement: Supplementary file 1 [file ijms-26-10616-s001.zip › ijms-3919406-supplementary.pdf]

Cytokine/Sample	SF CHS	SF OA	SF RA
IL-6	176.2±169.9	89.5±24.5 [43]	97.5±18.9 [43]
		277.4±368.7 [44]	225±221 [45]
TNF-α	423.6±806.9	2±3 [45]	4±3 [45]
		4±7 [46]	95±83 [46]
IL-10	9.128±7.893	35.4±16.5 [44]	40±37 [46]
TGF-β	669.7±452.2	583.2±38.92 [47]	9566±2331 [48]

Supplementary Table S1. Comparative cytokine quantification on synovial fluid (SF) from chronic hemophilic synovitis (CHS) patients versus osteoarthritis (OA) and rheumatoid arthritis (RA) conditions. SF samples of CHS patients at basal, before platelet-rich plasma (PRP) treatment, were compared with reported levels of IL-6 (43-45), TNF-α (45,46), IL-10 (44, 46) and TGF-β (47, 48) in OA or RA conditions. SF samples of CHS patients were centrifuged, and the acellular fraction (supernatant) was stored at –20 °C until analysis by ELISA, following the manufacturer's instructions for each analyte. Values are expressed as pg/mL ± standard deviation (SF from CHS, OA, and RA), before PRP treatment (n=8).

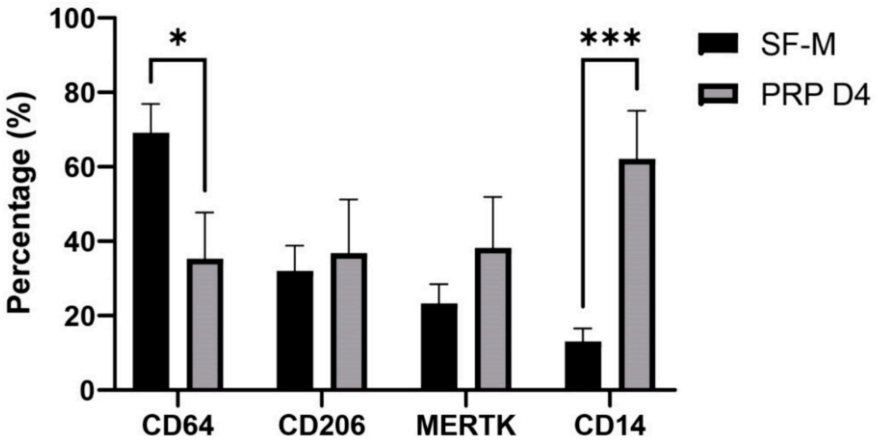

Supplementary Figure S1. Characterization of polarizing surface markers in monocyte-derived macrophages differentiated in the presence of SF or platelet-rich plasma (PRP). Flow cytometry analysis was performed to compare the percentage of M1 (CD64) and M2 (CD206, MERTK and CD14) markers in SF-induced (SF-M) versus PRP-induced macrophages. Two-way ANOVA test, followed by Sidak'k multiple comparisons test, SF-M D0 vs PRP D4 *p<0.05, ***p<0.001, n=6-12.

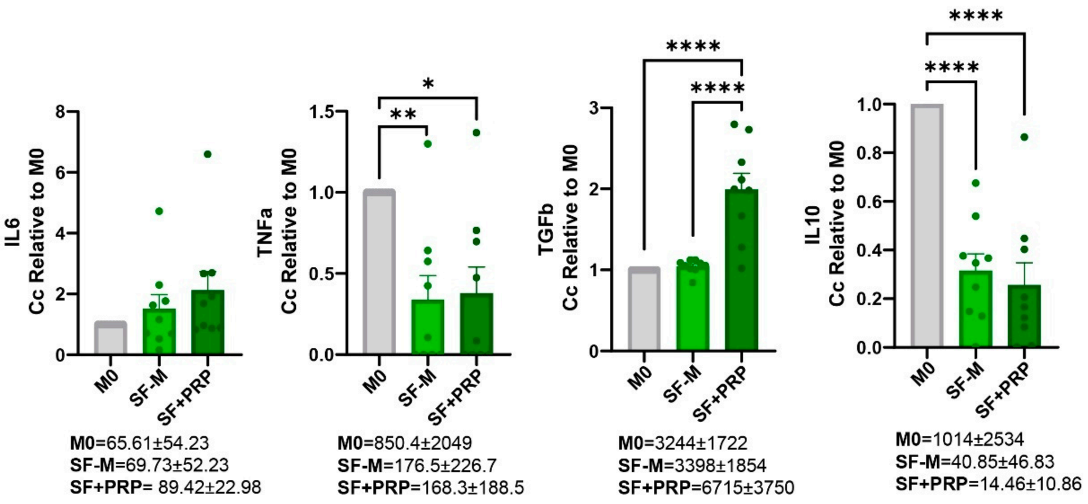

Supplementary Figure S2. Relative cytokine levels measured in the supernatant of SF-induced or SF+PRP-induced macrophages compared to M0. The quantification levels (pg/mL) of each cytokine was performed following the manufacturer's instructions for each analyte. Relative expression is indicated as fold changes of concentration (cc) referred to M0. Concentrations are also indicated below of each graph accompanied by the mean ± sd for each condition. *p<0.05, **p<0.01, ****p<0.001, Kruskal-Wallis test or one-way ANOVA and Dunn or Holm Sidák multiple comparisons test, n=9.

Cytokine/Sample	SF CHS	SF + PRP CHS
IL-6	176.2±169.9	261.2±274.8
TNF-α	423.6±806.9	209.4±355.5
IL-10	9.128±7.893	8.656±7.339
TGF-β	669.7±452.2	258.2±27.86

Supplementary Table S2. Cytokine quantification on SF from CHS patients before and after PRP treatment. SF samples from CHS patients were centrifuged, and the acellular fraction (supernatant) was stored at –20 °C until analysis by ELISA, following the manufacturer's instructions for each analyte. Samples include SF obtained either before PRP treatment or 2 weeks after the final PRP injection. Values are expressed as pg/mL ± standard deviation (SF from CHS, OA, and RA), before PRP treatment (n=8) and after PRP treatment (n=4).

A

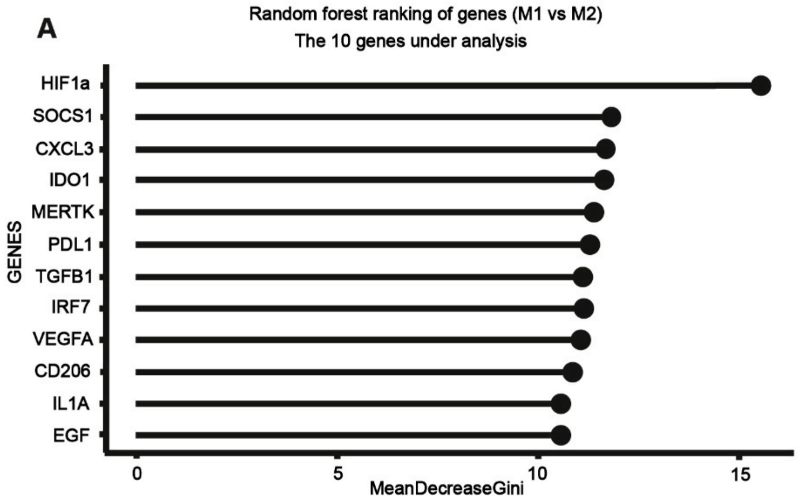

B

	% M1 <dlb>	% M2 <dlb>
N1 SF-M	75.6	24.4
N1 SF+PRP	60.2	39.8
N2 SF-M	71.4	28.6
N2 SF+PRP	56.6	43.4

Supplementary Figure S3. Gene Ranking and M1–M2 Prediction by Random Forest under SF and SF+PRP conditions. A) The genes evaluated by the algorithm were ranked based on the average decrease in the Gini index, indicating which of the genes is the best predictor of the model established to define the population under study as M1 (lower inequality or close to or equal to 0) or M1/M2 (higher inequality or close to or equal to 15) (n=3-11). B) Results of the prediction of the model comparing both SF-M and SF+PRP conditions vs the public database of RNA-seq from MoMacverse.

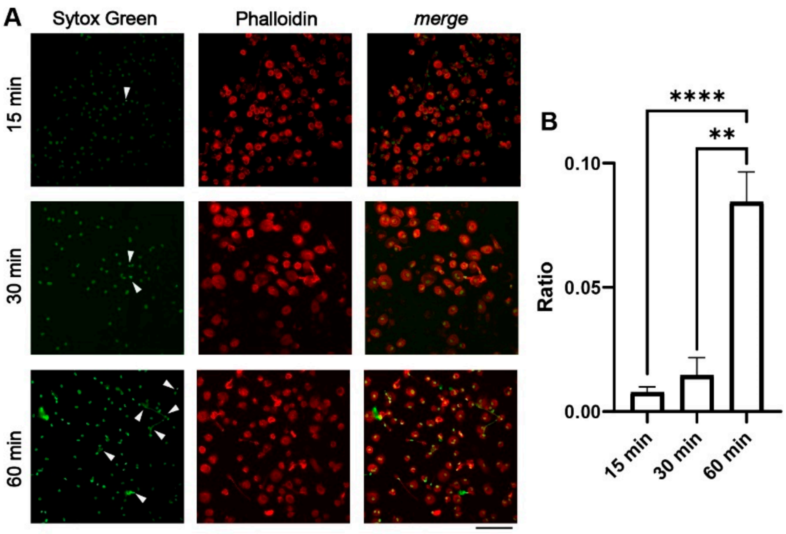

Supplementary Figure S4. Kinetics of macrophage phagocytosis of SF-induced ETs of CHS patients. A) NETs were induced using 10% SF from patients with CHS during 180 minutes. White arrows in the first panel (Sytox Green) indicate the presence of NETs at different time points. The second panel shows M0 macrophages stained with Phalloidin. In the third (merged) panel, co-localization can be observed. The phagocytosis of NETs was considered positive when at least part of the ETotic structure was visualized within the macrophage. Images were acquired by fluorescence confocal microscopy (20X, scale bar 20 µm). B) The phagocytosis ratio was calculated based on the number of cells containing ETotic structures within their cytosol, relative to the total number of cells per image. **p<0.01, **p<0.0001; Kruskal–Wallis test followed by Dunn's multiple comparisons test (n=2–3).

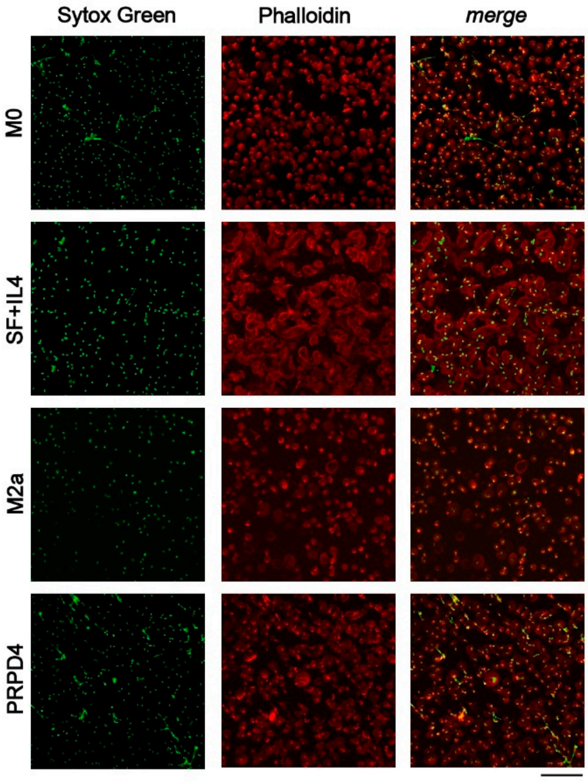

Supplementary Figure S5. NETs and type 2 signaling are not enough for giant multinucleated cell formation. MDM were obtained from healthy donors and incubated for 7 days, resulting in M0 (only basal medium), SF+IL4 (SF at day 0 and IL4 at day 4), M2a (IL4 at day 4) and PRPD4 (PRP at day 4). NETs were induced using 10% SF from patients with CHS for 180 minutes, and add to the conditions at day 7 for 60 minutes. The first panel (Sytox Green) indicate the presence of NETs, the second macrophages stained with Phalloidin and the Merged panel, co-localization can be observed. Images were acquired by fluorescence confocal microscopy (20X, scale bar 20 μm (n=2).
